# Azooxanthellate? Most Hawaiian black corals contain *Symbiodinium*

**DOI:** 10.1098/rspb.2010.1681

**Published:** 2010-10-20

**Authors:** Daniel Wagner, Xavier Pochon, Leslie Irwin, Robert J. Toonen, Ruth D. Gates

**Affiliations:** 1 Department of Oceanography, University of Hawai'i at Mānoa, 1000 Pope Road, Honolulu, HI 96822, USA; 2 Hawai'i Institute of Marine Biology, 46-007 Lilipuna Road, Kāne'ohe, HI 96744, USA; 3 University of Delaware, College of Earth, Ocean, and Environment, 111 Robinson Hall, Newark, DE 19716, USA

**Keywords:** precious coral, endosymbiosis, mesophotic coral reef ecosystems, Antipatharia, internal transcribed spacer-2

## Abstract

The ecological success of shallow-water reef-building corals (Hexacorallia: Scleractinia) is framed by their intimate endosymbiosis with photosynthetic dinoflagellates in the genus *Symbiodinium* (zooxanthellae). In contrast, the closely related black corals (Hexacorallia: Anthipatharia) are described as azooxanthellate (lacking *Symbiodinium*), a trait thought to reflect their preference for low-light environments that do not support photosynthesis. We examined 14 antipatharian species collected between 10 and 396 m from Hawai'i and Johnston Atoll for the presence of *Symbiodinium* using molecular typing and histology. *Symbiodinium* internal transcribed spacer-2 (ITS-2) region sequences were retrieved from 43 per cent of the antipatharian samples and 71 per cent of the examined species, and across the entire depth range. The ITS-2 sequences were identical or very similar to those commonly found in shallow-water scleractinian corals throughout the Pacific. Histological analyses revealed low densities of *Symbiodinium* cells inside antipatharian gastrodermal tissues (0–92 cells mm^−3^), suggesting that the *Symbiodinium* are endosymbiotic. These findings confirm that the capacity to engage in endosymbiosis with *Symbiodinium* is evolutionarily conserved across the cnidarian subclass Hexacorallia, and that antipatharians associate with *Symbiodinium* types found in shallow-water scleractinians. This study represents the deepest record for *Symbiodinium* to date, and suggests that some members of this dinoflagellate genus have extremely diverse habitat preferences and broad environmental ranges.

## 1. Introduction

Mesophotic coral reef ecosystems (MCEs) are coral reefs located below the depth limits of traditional scuba diving (40 m) and extend to the deepest portion of the photic zone, which may be over 150 m in tropical and subtropical regions with high water clarity [1–3]. MCEs are direct extensions of shallow-water reefs; however, because of their spatial separation from the many anthropogenic and natural stresses that affect shallow-water areas, MCEs have traditionally been considered a *de facto* refuge for the globally degraded shallow-water ecosystems [2–4]. Despite their importance, very little is known about the community structure of MCEs and the biological adaptations that allow mesophotic organisms to strive in low-light environments [2,3]. MCEs host a variety of photosynthetic organisms, such as algae and zooxanthellate corals, as well as non-photosynthetic organisms, like sclerosponges, azooxanthellate gorgonians and antipatharians [2]. The latter, commonly known as black corals, have been traditionally considered an exclusively azooxanthellate order of anthozoan hexacorals encompassing over 235 species [5,6], some of which are commercially harvested for the precious coral jewellery industry [7]. Antipatharians are primarily found in low-light environments, with approximately 75 per cent of the recognized species occurring deeper than 50 m [5]. The few species that occur in shallower water (less than 50 m) inhabit areas where light intensities are substantially reduced, such as inside caves or crevices, underneath overhangs, on steep vertical walls, or in areas with very high water turbidities [6–13]. Given the occurrence of antipatharians in dimly lit to dark areas in both shallow water (where they are present in shaded microenvironments) and deep water (where they are effectively shaded by decreasing light intensities with depth), the absence of *Symbiodinium* in this taxonomic group has generally been inferred, rather than empirically demonstrated. That said, close to a century ago several authors reported observing *Symbiodinium*-like entities in the tissues of various antipatharian species using microscopy [14–16]. More recent studies using histological techniques [8,17], spectrophotometric chlorophyll measurements [18,19] and molecular approaches [19] have, however, generally failed to confirm these early reports, with the exception of a single study reporting *Symbiodinium* in a shallow-water antipatharian (40 m) from Indonesia [6]. To address these inconsistencies in the literature and directly examine the symbiotic status of antipatharians, we used molecular and histological analyses to test for the presence of *Symbiodinium* in a wide taxonomic range of antipatharian corals collected in the waters surrounding Hawai'i and Johnston Atoll over a broad bathymetric gradient.

## 2. Material and methods

### (a) Sample collection

A total of 53 antipatharian samples belonging to 14 species, nine genera and five families were collected from the Hawaiian Islands and Johnston Atoll at depths between 10 and 396 m (electronic supplementary material, table S1). Samples included museum specimens deposited at the Bernice P. Bishop Museum in Honolulu, and specimens recently collected using scuba and the Hawai'i Undersea Research Laboratory (HURL) manned submersibles *Pisces* *IV* and *V* (electronic supplementary material, table S1). Collected specimens were (i) preserved in 95 per cent ethanol for molecular work, (ii) initially preserved in 10 per cent formalin in sea water and transferred to 70 per cent ethanol after 3–5 days for histological work, and (iii) frozen at −80°C for chlorophyll autofluorescence measurements.

### (b) Molecular data

Antipatharian samples were washed with distilled water, and 3–5 mm diameter tissue biopsies were removed and placed in 400 µl of guanidinium buffer (50% (w/v) guanidinium isothiocyanate; 50 mM Tris pH 7.6; 10 µM EDTA; 4.2 per cent (w/v) sarkosyl; 2.1 per cent (v/v) β-mercaptoethanol) at 4°C for one week. Samples were then placed at −80°C for 10 min, incubated at 72°C for 20 min and vortexed. Tubes were centrifuged at 14 000 rpm for 5 min and the resulting supernatant mixed 1 : 1 with isopropanol and incubated at −20°C overnight. Precipitated DNA was pelleted by centrifugation at 14 000 rpm for 15 min, the pellets washed in 70 per cent ethanol and resuspended in 50 µl Tris Buffer (0.1 M, pH 8). The DNA concentration and quality were assessed at 260 nm using a Nanodrop ND-1000, and the DNA stored at −20°C until analysed.

The internal transcribed spacer-2 (ITS-2) region was PCR-amplified using primers ‘ITS-DINO’ and ‘ITS2-REV2’, and conditions described by Pochon & Gates [20]. Positive PCR products were purified using the QIAquick PCR Purification Kit (Qiagen) and ligated into the pGEM-T Easy vector (Promega). Ten positive inserts per clone library were amplified using plasmid-specific (M13) primers and sequenced in both directions using the ABI Prism Big Dye Terminator Cycle Sequencing Ready Reaction Kit and an ABI 3100 Genetic Analyzer (Perkin Elmer-Applied Biosystems) at the University of Hawai'i.

The DNA sequences were inspected and assembled using Sequencher v. 4.7 (Gene Codes Corporation, Ann Arbor, MI, USA) and identified via BLAST comparisons in GenBank, as well as local BLAST to our in-house *Symbiodinium* databases. Alignments for clade C and clade D *Symbiodinium* were created using BioEdit v. 5.0.9 [21]. Each alignment was manually edited and only identical sequences recovered from two or more independent antipatharian samples included in the downstream analyses. Unconfirmed clone singletons were treated as artefacts or rare intragenomic repeats, and the nucleotide at the site of polymorphism was converted to the consensus following Stat *et al*. [22]. Each sequence variant was named following *Symbiodinium* ITS-2 nomenclature *sensu* LaJeunesse [23] (i.e. starting with a letter corresponding to the *Symbiodinium* clade and followed by a number referring to the within-clade diversity). Name extensions (b1, b2 or b3) were added to the novel sequence types. Sequences were then compared with all unique *Symbiodinium* ITS-2 sequences deposited in GenBank as of April 2010, resulting in alignments of 390 sequences for clade C and 49 sequences for clade D. Owing to the polytomic nature of *Symbiodinium* ITS-2 sequences within clade C, unrooted phylogenetic inferences were generated using the neighbour-joining method implemented in the program Mega v. 4.0 [24], with the p-distance model and the gaps treated as pairwise deletions. GenBank accession numbers of all sequences included in our analyses are indicated in electronic supplementary material, figures S1 and S2.

### (c) Histology

Antipatharian samples from which *Symbiodinium* ITS-2 sequences were recovered were processed for histological analysis. Tissues were removed from the skeleton via dissection and dehydrated by sequential submersions in 70 per cent ethanol for 30 min, 95 per cent ethanol for 1.5 h and 100 per cent ethanol for 6 h, followed by clearing in xylene for 4 h. Samples were infiltrated with molten paraffin wax at 70°C for 16 h and poured into standard moulds. Serial histological sections were cut at 5 µm using a Leica RM 2155 rotary microtome. Sections were stained with Masson's Trichrome using the following staining steps: xylene for 5 min, 100 per cent ethanol for 5 min, haematoxylin Z for 25 min, tap water wash for 15 min, phosphomolybic acid for 5 min, light green for 5 min, 100 per cent ethanol for 5 min, 100 per cent ethanol for 2 min and xylene for 5 min. Sections were viewed under an Olympus BX51 compound microscope with a camera attachment.

### (d) Chlorophyll autofluorescence

Samples of two antipatharian species for which fresh samples could be obtained (*Antipathes griggi* and *Cirrhipathes* cf. *anguina*) were processed for chlorophyll autofluorescence. Frozen samples were left to thaw at room temperature. Tentacle tissues were dissected and placed onto microscope slides and viewed under an Olympus Fluoview 1000 laser scanning confocal microscope using a blue light excitation at 430–470 nm, followed by emission through a 500–530 nm long-pass filter.

## 3. Results

**Figure 1.**
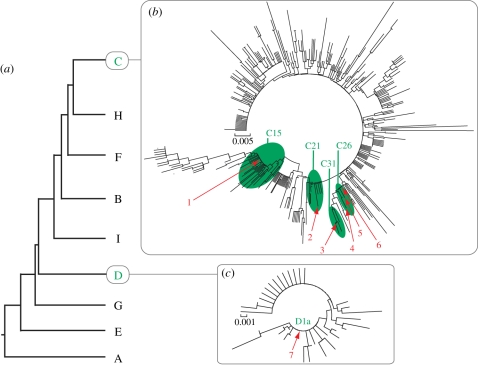
*Symbiodinium* ITS-2 sequence types recovered from antipatharian species. (*a*) Sequences belong to two of nine clades that comprise the genus *Symbiodinium* (cladogram modified from Pochon & Gates [20]). Unrooted circled trees of (*b*) 390 ITS-2 sequences in *Symbiodinium* clade C and (*c*) 49 ITS-2 sequences in *Symbiodinium* clade D. Sequences were identical or closely related to *Symbiodinium* sequence types C15, C21, C26, C31 and D1a. Numbers correspond to specific ITS-2 sequences (1, C15; 2, C21_b1; 3, C31; 4, C26_b3; 5, C26_b2; 6, C26_b1; 7, D1a). Scale bars correspond to the number of changes per site (see electronic supplementary material, figures S1 and S2 for details).

*Symbiodinium* were identified by genotyping the ITS-2 region of the nuclear ribosomal cistron and exploring their location in the tissues of a subset of corals using histology and chlorophyll autofluorescence. *Symbiodinium* ITS-2 sequences were isolated from 23 of the 53 antipatharians sampled (43 per cent), representing individuals collected across the entire surveyed depth range (10–396 m) and 10 of the 14 species examined (table 1). A total of 219 ITS-2 *Symbiodinium* sequences were recovered, six from clade C and one from clade D. Four of the six clade C sequences were novel (figure 1), and three of these (C21_b1, C26_b2 and C26_b3) were the most commonly encountered sequences in the dataset, being recovered 62, 45 and 67 times, respectively (electronic supplementary material, table S2). These novel clade C sequences were extremely closely related to *Symbiodinium* C21 and C26, differing by only one or two bases (figure 1). The two known clade C sequence types were C15 and C31, types previously described as the dominant endosymbionts in the scleractinian coral genera *Porites* and *Montipora*, respectively, from Hawai'i and the broader Pacific [22,23,25]. A low number of *Symbiodinium* D1a sequences were retrieved from *A. griggi* collected at 24 m and *Stichopathes* sp. collected at 350 m (table 1).

**Table 1.** Antipatharian samples in which *Symbiodinium* were detected using ITS-2 genotyping and histology. n.a., not applicable.

species	family	colonies sampled	colonies containing *Symbiodinium*	depth (m)	ITS-2 sequences	*Symbiodinium* density (cells mm^−3^)
*Cirrhipathes* cf. *anguina*	Antipathidae	5	3	11–30	C21_b1, C26_b1, C26_b2, C26_b3	0–40.8
*Antipathes griggi*	Antipathidae	8	1	24	C21_b1, C26_b1, D1a	0–92
*Antipathes grandis*	Antipathidae	10	3	34–91	C21_b1, C26_b1, C26_ b3	0–25
*Aphanipathes* sp.	Aphanipathidae	10	4	88–127	C15, C21_b1, C26_b1, C26_b2, C26_b3	0–14.8
*Myriopathes ulex*	Myriopathidae	3	1	96	C15	n.a.
*Stichopathes* cf. *echinulata*	Antipathidae	6	3	129	C15, C21_b1, C26_b1, C26_b2, C26_b3	0–2.9
*Stichopathes* sp.	Antipathidae	5	5	182–396	C21_b1, C31, C26_b1, C26_b2, C26_b3, D1a	n.a.
*Acanthopathes undulata*	Myriopathidae	1	1	259	C26_b1, C26_b2	n.a.
*Bathypathes* sp.	Schizopathidae	1	1	320	C26_b3	n.a.
*Myriopathes*? sp.	Myriopathidae	1	1	396	C15	n.a.

**Figure 2.**
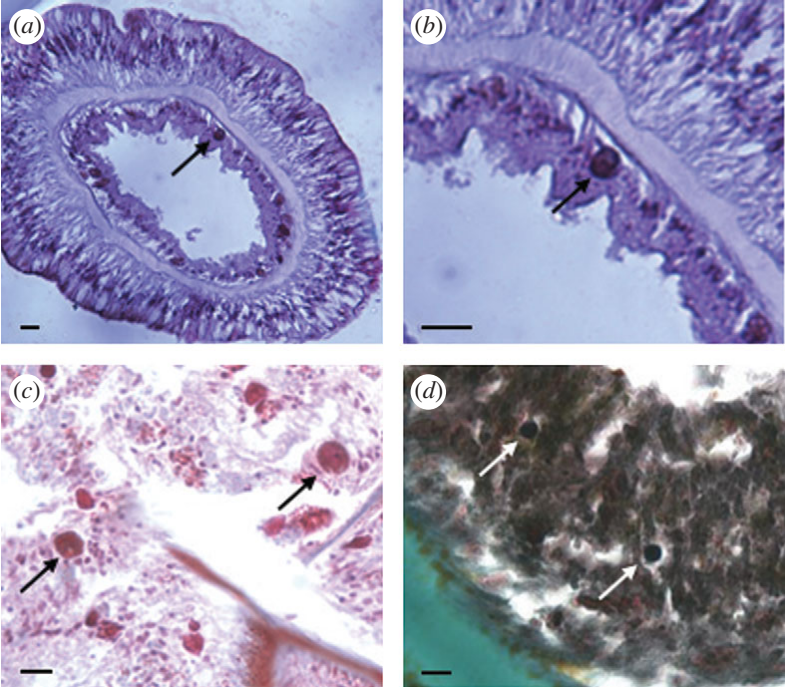
*Symbiodinium*-like cells inside the gastrodermal cells of various antipatharian hosts. (*a*,*b*) Cross-section through the tentacle of *A. griggi*, (*c*) longitudinal section through the body cavity of *Antipathes grandis* and (*d*) longitudinal section through the tentacle of *C.* cf. *anguina* (scale bars = 10 µm; arrows point to *Symbiodinium*-like cells).

Serial histological sections showed *Symbiodinium*-like cells at very low densities (0–92 cells mm^−3^) inside gastrodermal cells in the tentacles and body cavities of five antipatharian species (figure 2 and table 1). The *Symbiodinium*-like cells were 5.01–11.45 µm in diameter and patchily distributed within the antipatharian tissues, with most sections containing none. Oocytes of various stages of maturity were also observed along the primary transverse mesenteries in all examined antipatharian species; however, none of them contained *Symbiodinium*. Chlorophyll autofluorescence was observed at locations that corresponded to the position of *Symbiodinium*-like cells in the gastrodermal tissues of the two antipatharian species for which fresh samples could be obtained (data not shown).

## 4. Discussion

Black corals have traditionally been assumed to be azooxanthellate because they are generally found in low-light environments that do not support photosynthesis [5–13]. Our data provide evidence that antipatharians serve as habitat for *Symbiodinium* types that are commonly associated with some shallow-water scleractinian corals. *Symbiodinium* ITS-2 sequences were retrieved from 43 per cent of the antipatharian samples examined and 71 per cent of the species surveyed. All retrieved sequences were either identical or closely related to those commonly found in some scleractinian corals from the Pacific. *Symbiodinium* C15 is the dominant endosymbiont of *Porites* spp., and C21, C31 and C26 are all found in endosymbiotic communities hosted by *Montipora* spp. [22,23,25]. We also detected rare occurrences of *Symbiodinium* D1a, an opportunistic and thermally tolerant *Symbiodinium* type [26] previously found in a broad range of hosts and geographical locations, whose abundance increases on reefs during and after episodes of severe bleaching and mortality [27,28]. These findings collectively suggest that these *Symbiodinium* types exist over extremely broad environmental ranges and have very diverse habitat preferences.

Histological analysis of antipatharian samples confirmed the presence of *Symbiodinium*-like cells inside antipatharian gastrodermal tissues (figure 2 and table 1). These observations provide evidence that the recovered *Symbiodinium* ITS-2 sequences represent endosymbionts rather than *Symbiodinium* cells associated with the surface, or trapped in the guts of the antipatharians. Additionally, none of the observed antipatharian oocytes contained *Symbiodinium*, suggesting that antipatharian hosts acquire endosymbionts horizontally from their environment and not vertically from the parent. Furthermore, there was no pattern in the recovery of particular *Symbiodinium* types from individuals of the same antipatharian species (electronic supplementary material, table S2), suggesting that endosymbiont acquisition occurs opportunistically and is not host-specific. The low densities of *Symbiodinium* cells inside antipatharian gastrodermal tissues (0–92 cells mm^−3^), coupled with the extreme depths at which they were detected here (≤396 m), suggests that they are unlikely to fix carbon or play a significant role in the nutrition of their antipatharian hosts. This interpretation is consistent with the fact that *Symbiodinium* were detected here below the compensation depth for photosynthesis, and with other studies showing that zooxanthellate corals rely more heavily on heterotrophic feeding than on autotrophy at increasing depths [29,30]. Whether a shift towards coral heterotrophy is associated with a concomitant shift in the interactive status of *Symbiodinium* from mutualist to commensal or parasite was not determined in the latter studies [29,30], although a range of interactive physiologies has been hypothesized to exist in *Symbiodinium* endosymbioses with corals [31]. The low densities of *Symbiodinium* in antipatharian tissues, coupled with the extremely low-light conditions at the depths where many of the samples were collected here, do not support the interpretation that the association is a nutritionally framed mutualism. Future research will be needed to define the fitness benefits and trade-offs of hosting *Symbiodinium* in these deep environments, and to determine whether *Symbiodinium* survive there by exploiting rather than benefiting their hosts.

A striking outcome of this study is the extreme depths at which *Symbiodinium* were recorded (table 1), at least some of which is below the compensation depth for photosynthesis in Hawaiian waters (approx. 125 m) [32]. These findings suggest that the carbon demand of these dinoflagellates is either reduced by dormancy or met by means other than photosynthesis, such as by self-digestion or by heterotrophic feeding on an external carbon source. Heterotrophic endosymbiont feeding has been suggested in zooxanthellate invertebrates that are seasonally exposed to environmental conditions that do not support photosynthesis [33,34], and such modes of nutrition would make sense for *Symbiodinium* populations that are located below the compensation depth for photosynthesis. Additional research will be needed in order to elucidate the physiological roles of *Symbiodinium* populations located in such low-light environments that do not support net photosynthesis.

Our identification of *Symbiodinium* inside the tissues of multiple antipatharian taxa (table 1) confirms that endosymbiosis with *Symbiodinium* is evolutionarily conserved across the cnidarian subclass Hexacorallia. This group encompasses six taxonomic orders (Actiniaria, Antipatharia, Ceriantharia, Corallimorpharia, Scleractinia and Zoantharia) and over 4300 extant species, found in very diverse habitats [35]. Until now, widespread *Symbiodinium* associations had been reported in all hexacorallian orders with the exception of the Antipatharia [36]. Here we demonstrate that this exception reflects the historical constraints of sampling a taxonomic group that primarily resides in deep-water environments [5]. With attention and sampling effort, other important discoveries are likely to be made about the basic biology of organisms that inhabit these important and understudied deep-water ecosystems.

The broad depth range over which *Symbiodinium* were detected here (10–396 m) suggests that at least some members of this dinoflagellate genus have incredibly diverse habitat preferences and broad environmental ranges. The previous deepest record for *Symbiodinium* is in an unidentified madreporarian coral at 200 m off southern Florida [36,37]. *Symbiodinium* were recorded here in several antipatharian samples collected below this depth and down to 396 m (table 1), thus extending the known bathymetric range for *Symbiodinium*. Although these deep records do not represent expansions in the known temperature ranges of these dinoflagellates, which have previously been reported to survive at temperatures as low as 3°C in surface waters off southern New England [34], they highlight that sampling effort for *Symbiodinium* has historically been low in deeper waters. Advances in deep diving technologies (mixed-gas technical diving, manned submersibles, remotely operating vehicles, etc.) have only recently enabled more thorough investigations of habitats below the depth limits of traditional scuba diving, but our functional understanding of these deeper ecosystems and the basic biology of the organisms that inhabit these systems is still marginal [2–4]. In conclusion, this study indicates that antipatharians serve as habitat for *Symbiodinium* types found in some shallow-water scleractinians and that the capacity to engage in endosymbiosis with *Symbiodinium* is evolutionarily conserved across the cnidarian subclass Hexacorallia. This study also represents the deepest record for *Symbiodinium* to date, and suggests that some members of this dinoflagellate genus have incredibly broad habitat preferences and environmental ranges. Lastly, this study highlights how little is known about the basic biology of corals found below the depths accessible through traditional scuba diving.
